# Nutrition and Quality of Life for Patients with Chronic Disease

**DOI:** 10.3390/nu17132170

**Published:** 2025-06-30

**Authors:** Evridiki Patelarou, Konstantinos Giakoumidakis

**Affiliations:** Department of Nursing, School of Health Sciences, Hellenic Mediterranean University, 71410 Heraklion, Greece; epatelarou@hmu.gr

## 1. Introduction

Chronic conditions such as cancer, heart disease, metabolic syndrome, liver disease, and renal failure significantly influence patients’ social, emotional, and functional well-being, in addition to their physical symptoms [1,2]. Among the many modifiable aspects of health in chronic illness, nutrition has a significant impact on both overall quality of life (QoL) and clinical outcomes [3]. It appears that targeted nutrition interventions can modify immune and inflammatory responses, which are crucial parameters in the development of chronic disease [4]. Additionally, personalized nutrition therapy enhances the clinical course of patients with a wide range of chronic diseases, enhancing muscle functionality and fostering recovery mechanisms [5,6].

This Special Issue of *Nutrients*, titled “Nutrition and Quality of Life for Patients with Chronic Disease,” brings together a multidisciplinary collection of original research and thorough reviews that clarify how individualized nutritional approaches can change the course of many chronic diseases, enhance functional ability, and increase life satisfaction. The nine published articles, which span the fields of hepatology, nephrology, rheumatology, cancer, cardiovascular health, public health, and public policy, demonstrate the complexity and potential of nutrition-based interventions in improving patient care. Apart from physical health, social and emotional domains, including social isolation and behavioral compliance, are also important factors influencing patient outcomes and health-related quality of life [7]. These behavioral interventions, which can help maintain lifestyle and dietary changes, are key to long-term adherence to dietary recommendations, which emphasizes the need for interdisciplinary care when dealing with chronic disease [8,9].

## 2. Key Contributions

Perlinski and Sobocki [10] thoroughly investigated advanced malignancy in chronic intestinal failure, home parenteral nutrition (HPN), and malignant bowel obstruction. Their pooled analysis of 34 studies showed that, with appropriate management, HPN can enhance QoL and, in some cases, prolong survival. Positive outcomes were associated with higher albumin levels, better performance status, and continued treatment. In the meantime, the authors emphasized the significant psychological effects of chronic HPN and supported patient-centered care strategies that combine nutritional therapy with mental health and social support.

Ciaffi et al.’s [11] scoping review of 19 studies explained the detrimental effects of ultra-processed foods (UPFs) on bone health and joint diseases, particularly in postmenopausal women and patients with knee osteoarthritis. UPF consumption was linked to an increased risk of osteoporosis, reduced bone mineral density, and a higher incidence of gout and rheumatoid arthritis. These results call for immediate public health measures to lower UPF consumption and encourage musculoskeletally appropriate whole-food eating habits.

A qualitative study of advanced liver disease identifies several intricate causes of malnutrition, including gaps in healthcare delivery, dietary and physical limitations, symptom burden, a lack of social support, and structural issues [12]. The shortcomings of the current screening tools, which fail to adequately capture psychological and systemic factors, are revealed through interviews with doctors, caregivers, and patients. This leads to recommendations for comprehensive, customized screening techniques that will more effectively detect and address nutritional risk.

Notarnicola et al. [13] present compelling evidence from a randomized controlled study showing that daily orange consumption significantly reduces liver steatosis in metabolic dysfunction-associated steatotic liver disease. Individuals consuming 400 g of oranges per day showed a 30% decrease in liver fat after just four weeks, regardless of weight loss, suggesting the direct hepatoprotective effect of phytochemicals. The study supported the inclusion of foods high in phytochemicals in therapeutic diets for liver illnesses, even though fibrosis remained unchanged.

In a mouse model, experimental data from Hu et al. [14] showed that maize protein hydrolysate fermented by lactic acid bacteria (FCH) has anti-fatigue properties. High-dose FCH improved endurance, decreased oxidative stress, restored liver and muscle glycogen, and changed the ecology of the gut by raising Lactobacillus abundance. These results point to a potential gut–muscle axis mechanism and support further research on FCH as a functional meal with the goal of reducing fatigue.

To treat metabolic syndrome in low-resource settings, Alcaide-Leyva et al. created a highly sensitive and reasonably priced diagnostic methodology specifically for an urban population in the Peruvian Amazon [15]. The model achieved over 90% sensitivity in detecting metabolic syndrome in a population with a nearly 48% prevalence using systolic blood pressure and very-low-density lipoprotein cholesterol, highlighting the need for population-specific tools to guide targeted public health interventions in rapidly urbanizing settings.

According to the Spanish researchers García Samuelsson et al. [16], even if they do not have any overt signs of metabolic syndrome, metabolically healthy obese (MHO) people are more likely to develop insulin resistance. Low levels of physical activity, poor adherence to the Mediterranean diet, and socioeconomic disparities were identified as the primary causes of metabolic deterioration in a cohort of 68,884 obese workers, underscoring the transient nature of the MHO phenotype and the urgent need for early lifestyle interventions, particularly in occupational health settings.

An eight-year Indonesian cohort study by Retiaty et al. [17] examined the relationship between rising central and body mass index-based obesity rates and changing dietary patterns, which were characterized by a high intake of fat and oil and a low intake of fiber. Interestingly, despite increasing obesity rates, the biomarkers for non-communicable diseases remained relatively stable, highlighting the intricate connection between diet, obesity, and metabolic health, and pointing to important areas for intervention.

Finally, our research team translated and validated the Greek version of the Cardiovascular Diet Questionnaire 2 (CDQ-2), providing a validated tool for assessing the dietary habits of patients with cardiovascular disease. The Greek version of the CDQ-2 demonstrated high reliability and strong agreement with the current Mediterranean Diet Adherence Screener (MEDAS) measure, particularly among smokers, younger adults, men, and those who were less physically active. This proven technique provides a useful way to evaluate and adjust dietary interventions in cardiovascular disease management [18].

## 3. Conclusions

This Special Issue underscores the central role of nutrition in the comprehensive management of chronic diseases, addressing not only physical symptoms, but also mental health and overall quality of life. The studies collectively demonstrate how targeted dietary interventions—ranging from functional food compounds and validated screening tools to lifestyle modifications and micronutrient therapies—can prevent metabolic deterioration, support organ integrity, and alleviate disease burden. These findings advocate for integrated, individualized care models that address the biological, psychological, and social dimensions of nutrition-related health, recognizing the need for both clinical precision and cultural relevance [19,20]. Future research should adopt longitudinal, interdisciplinary designs to enhance risk stratification, unravel the mechanisms of nutrition–disease interactions, and scale effective nutritional care models—from clinical practice to occupational, community, and public health settings. Building stronger teamwork among healthcare professionals is key to successfully putting these care models into practice [21]. Ultimately, advancing nutrition as a pillar of evidence-based clinical practice holds the potential to transform the lives of millions living with chronic diseases worldwide [22].
